# Atezolizumab plus bevacizumab in patients with unresectable or metastatic mucosal melanoma: 3‐year survival update and multi‐omics analysis

**DOI:** 10.1002/ctm2.70169

**Published:** 2025-01-05

**Authors:** Jie Dai, Tianxiao Xu, Lifeng Li, Meiyu Fang, Jing Lin, Jun Cao, Xue Bai, Caili Li, Xiaoting Wei, Junjie Gu, Yaoyao Liu, Xuan Gao, Xuefeng Xia, Jun Guo, Yu Chen, Lili Mao, Lu Si

**Affiliations:** ^1^ Key Laboratory of Carcinogenesis and Translational Research (Ministry of Education/Beijing), Department of Melanoma and Sarcoma Peking University Cancer Hospital and Institute Beijing China; ^2^ Department of Urology Second Hospital of Tianjin Medical University Tianjin China; ^3^ Geneplus‐Beijing Beijing China; ^4^ Department of Rare Cancer & Head and Neck Medical Oncology Cancer Hospital of the University of Chinese Academy of Sciences, Key Laboratory of Head & Neck Cancer Translational Research of Zhejiang Province Hangzhou China; ^5^ Department of Medical Oncology Fujian Cancer Hospital & Fujian Medical University Cancer Hospital Fujian China; ^6^ Key Laboratory of Carcinogenesis and Translational Research (Ministry of Education/Beijing), Department of Genitourinary Oncology Peking University Cancer Hospital and Institute Beijing China

**Keywords:** atezolizumab, bevacizumab, immunotherapy, mucosal melanoma, VEGF inhibitor

## Abstract

**Background:**

Atezolizumab plus bevacizumab has shown promising efficacy in advanced mucosal melanoma in the multi‐centre phase II study. This report updates 3‐year survival outcomes and multi‐omics analysis to identify potential response biomarkers.

**Methods:**

Forty‐three intention‐to‐treat (ITT) patients received intravenous administration of atezolizumab and bevacizumab every 3 weeks. Available samples underwent whole exome sequencing, transcriptome sequencing and targeted bisulphite sequencing to assess correlations with clinical outcomes.

**Results:**

With a median follow‐up of 40.3 months, the median overall survival (mOS) was 23.7 months (95% confidence interval [CI], 15.1–34), and the 3‐year OS rate was 28.7% (95% CI, 17.6%–46.8%). Patients with upper site melanoma exhibited longer progression‐free survival (PFS), higher tumour neoantigen burden (TNB) and greater copy number variations (CNVs) burden compared to those with lower site melanoma. *NRAS* mutations were associated with enhanced angiogenesis, with five of six patients achieving partial response. Inflammatory cell infiltration, angiogenic status and activation of the SMAD2 and p38 MAPK pathways may be prognostic indicators.

**Conclusions:**

This 3‐year updated analysis confirms the sustained efficacy of atezolizumab in combination of bevacizumab in patients with advanced mucosal melanoma. Inflammatory cell infiltration and angiogenic status were associated with therapeutic response. Furthermore, mucosal melanoma of upper site and *NRAS* mutation appear to be good predictors of response to immune checkpoint inhibitor and anti‐angiogenic combination treatment. Targeting SMAD2 and p38 MAPK pathways may further improve the outcome of mucosal melanoma.

**Key points:**

- 3‐year follow‐up study confirmed the therapeutic efficacy of atezolizumab combined with bevacizumab
- Tumors in the upper site and NRAS mutations are more sensitive to treatment
- Inflammatory cell infiltration, angiogenic status, and activation of the SMAD2 and p38 MAPK pathways may be prognostic indicators

## Graphical abstract

1. After a 3‐year follow‐up, this study further confirms that the combination therapy of atezolizumab and bevacizumab has long‐term efficacy in mucosal melanoma.
2. Patients with upper site melanoma and NRAS mutation exhibited a more favourable response to this combination treatment.
3. Inflammatory cell infiltration, angiogenic status and activation of the SMAD2 and p38 MAPK pathways may be prognostic indicators.

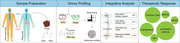

## 1 INTRODUCTION

Mucosal melanoma is an aggressive subtype of melanoma, and the common primary locations include nasal and oral cavity, gastrointestinal and urinary tract and gynaecological tissue.^1^ Mucosal melanoma represents only about 1.3% of all melanoma cases in Caucasian populations,^2^ but accounting for about 25% of all melanoma cases.^3^, ^4^, ^5^, ^6^ The aetiology and pathogenesis of mucosal melanoma are still not clearly defined. Mucosal melanoma has a distinctly different genomic landscape^7^, ^8^ compared to cutaneous melanoma, which is characterised by a markedly lower mutation burden and a predominance of large‐scale chromosomal structural variants. This unique genomic landscape is associated with a poorer prognosis^1^, ^3^ and diminished response to standard treatments for cutaneous melanoma, such as BRAF inhibitors.^8^, ^9^, ^10^ Consequently, there is an urgent demand for the exploring of new and effective therapeutic strategies for mucosal melanoma.

Immune checkpoint inhibitors (ICIs) are now recommended as the standard treatments in advanced mucosal melanoma, although the data showed a lower clinical efficacy when compare with that in cutaneous melanoma.^11^, ^12^ The objective response rate (ORR) of mucosal melanoma to anti‐PD‐1/PD‐L1 monotherapy was 0%–23.3%,^11^, ^12^, ^13^ which was inferior to that of cutaneous melanoma (33.7%–43.7%).^14^, ^15^ Combination ICIs with treatments targeting other mechanisms of immune evasion maybe a promising strategy to exert greater therapeutic effects. Vascular endothelial growth factor (VEGF), an essential regulator of angiogenesis, is crucial in driving melanoma progression.^16^, ^17^ It suppresses anti‐tumour immunity and reduces the effectiveness of ICI treatments by enhancing the pro‐tumour activity of immunosuppressive cells.^18^, ^19^ Multiple clinical studies have showed that dual targeting VEGF and PD‐1/PD‐L1 pathways could significantly improve the clinical outcomes in metastatic renal cancer,^20^, ^21^ non‐small cell lung cancer,^22^, ^23^ and hepatocellular carcinoma.^24^ In 2019, we initiated a multi‐centre phase II study combining atezolizumab with bevacizumab in patients with advanced mucosal melanoma (NCT04091217). The result demonstrated promising responses with an ORR of 45%, with a median progression‐free survival (mPFS) of 8.2 months, and a median duration of response of 12.5 months.^25^

Given most of patients with mucosal melanoma could not benefit from current immunotherapies, elucidation of the molecular basis of clinical heterogeneity is needed to deepen our understanding of resistance mechanisms to immunotherapy and select the optimised treatment modalities. It has been reported that several factors are associated with response to dual ICIs and VEGF inhibitor treatment, including angiogenesis, PD‐L1 expression, T‐cell response and myeloid inflammation signature in renal cancer and hepatocellular carcinoma.^26^, ^27^ However, potential predictive biomarkers for patients with mucosal melanoma are still limited. Here, we evaluated the genome, transcriptome and methylation signatures of tumours from the patients enrolled in the NCT04091217 clinical trial, and classified them into different molecular subgroups which may predict the clinical outcomes and further improve the development of combination individualised treatments for patients with mucosal melanoma.

## 2 RESULTS

### 2.1 Study population and survival updates

**FIGURE 1:**
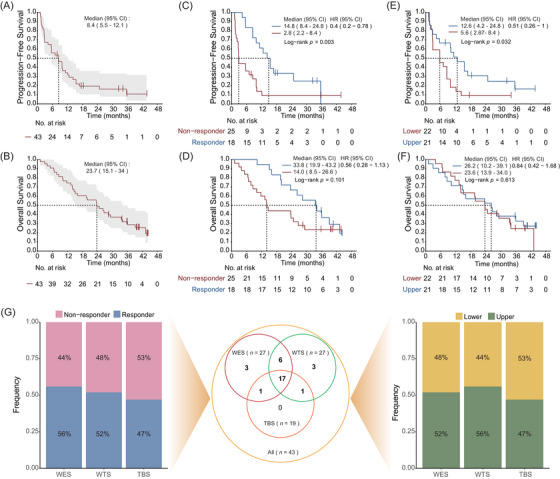
Study population and Kaplan–Meier estimates of progression‐free survival (PFS) and overall survival (OS). (A, B) Kaplan–Meier curve for PFS (A) and OS (B) of 43 intention‐to‐treat (ITT) patients. (C, D) Kaplan–Meier estimates of PFS (C) and OS (D) stratified by efficacy group (non‐responder [*n* = 25] and responders [*n* = 18]). (E, F) Kaplan–Meier analyses of PFS (E) and OS (F) for patients stratified by the primary site (lower sites [*n* = 22] and upper sites [*n* = 21]). (G) The Venn plot showing the number of samples that underwent whole exome sequencing (WES) and whole transcriptome sequencing (WTS), and targeted bisulphite sequencing (TBS). The bar chart represents the distribution of non‐responder/responder and lower/upper primary site in each analysis group. CI, confidence interval; HR, hazard ratio.

In the clinical trial, from 20 November 2019 to 3 December 2020, a total of 43 patients were administered atezolizumab plus bevacizumab and were subsequently included in the intention‐to‐treat (ITT) population. The patient demographics and baseline characteristics have been reported previously^25^ (Table S1). As of 15 August 2023, the median follow‐up duration was 40.3 months (interquartile range, IQR: 34.7–43.6). The mPFS was 8.4 months (95% confidence interval [CI], 5.5–12.1); the 1‐, 2‐ and 3‐year PFS rates were 34.0% (95% CI, 22.3%–51.9%), 19.5% (95% CI, 10.5%–36.1%) and 10.8% (95% CI, 3.7%–31.6%), respectively (Figure 1A). The median overall survival (mOS) was 23.7 months (95% CI, 15.1–34), with 1‐, 2‐ and 3‐year OS rates of 74.4% (95% CI, 62.5%–88.7%), and 48.8% (95% CI, 36%–66.3%), 28.7% (95% CI, 17.6%–46.8%), respectively (Figure 1B). The mPFS for responders was 14.8 months (95% CI, 8.4–24.8), which was significantly longer than that of non‐responders (median: 2.8 months, 95% CI, 2.2–8.4; hazard ratio, HR = .4, 95% CI, .2–.78; *p *= .003; Figure 1C). Responders had numerical favourable OS compared to non‐responders (median: 33.8 months, 95% CI, 19.9–43.2, vs. 14.0 months, 95% CI, 8.5–26.6 months; HR = .56, 95% CI, .28–1.13; *p *= .101; Figure 1D). The mPFS of upper sites was longer than lower sites (median: 12.6 months, 95% CI, 4.2–24.8 vs. 5.6 months, 95% CI, 2.87–8.4; HR = .51, 95% CI, .26–1; *p *= .032; Figure 1E), but the OS was similar between upper sites and lower sites (HR = .84, 95% CI, .42–1.68; *p *= .613; Figure 1F).

Among all 43 patients enrolled in the clinical trial, 31 patients with available tumour samples underwent multi‐omics sequencing. Based on the rigorous quality control (QC), 27 samples passed the QC for whole exome analyses, 27 for transcriptome analyses and 19 for DNA methylation analyses (Figure 1G). The sample numbers of responses (responders and non‐responders) and primary tumour sites (upper and lower) were approximately equal across each sequencing cohort.

### 2.2 Genomic comparison of responders and non‐responders identifies response‐related genomic characters

**FIGURE 2:**
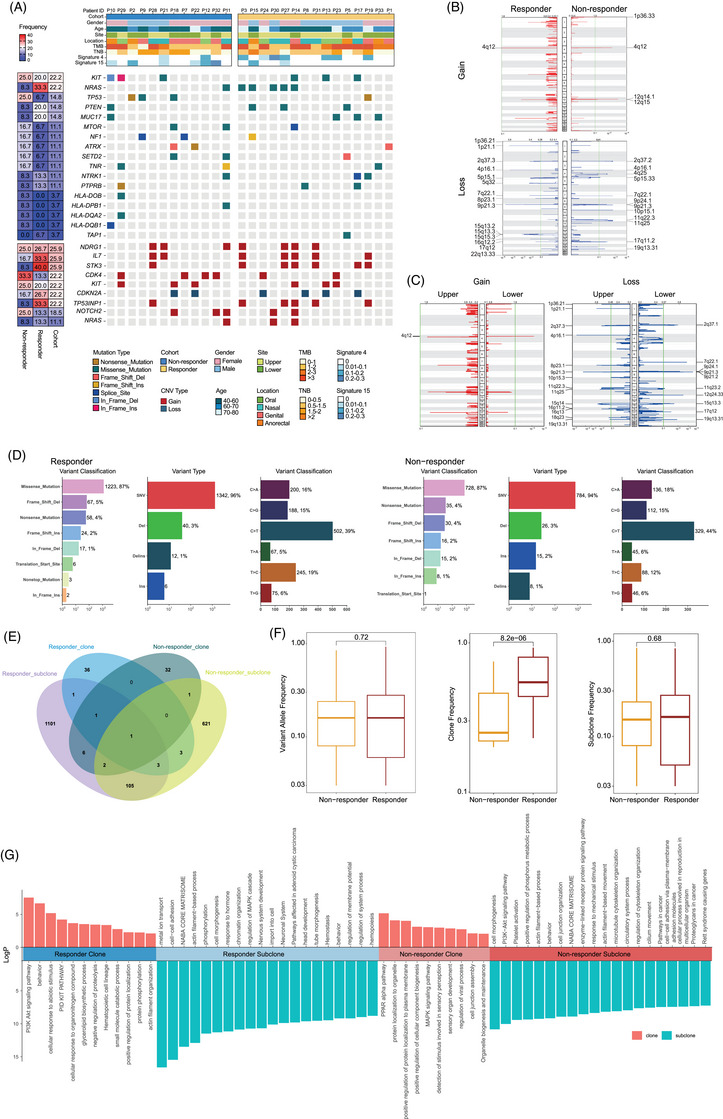
Overview of clinical and genetic characteristics in baseline tumours of non‐responders and responders to the atezolizumab plus bevacizumab therapy. (A) Top panel displays clinical features of non‐responder (*n* = 12) and responder (*n* = 15) samples underwent genomic sequencing, including patient ID, gender, age and primary site locations. Tumour mutational burden (TMB), tumour neoantigen burden (TNB) and mutational signature are also shown. The middle panel presents the oncoplot of common oncogenic driver genes and genes associated with antigen presentation. The bottom panel depicts the copy number variations (CNVs) in indicated genes. (B, C) GISTIC plots of genomic regions with copy number gain or loss in patients stratified by efficacy group (B) and primary sites (C). Red represents CNV gain, and blue represents CNV loss. (D) Bar plot of the distribution of mutation spectra, including variant classifications, variant types and single nucleotide variations (SNVs) in responders and non‐responders. (E) The Venn plot showing the number of shared clones and subclones between the two groups. (F) Comparison of the frequencies of variant alleles, clones and subclones between the two groups. (G) Gene enrichment pathways of clonal and subclonal variants are shown.

The genomic landscape of responders and non‐responders is illustrated in Figure 2A. Tumour mutation burden (TMB) of our cohort ranged from .9 to 11.6 non‐synonymous mutations/megabase (mut/Mb). No significant differences in TMB (*p *= .329), tumour neoantigen burden (TNB; *p *= .478) or copy number variations (CNVs) burden (*p *= .901) were observed between two groups (Figure S1A–C). The TMB was comparable between upper and lower sites (*p *= .526), but the upper sites had higher TNB (*p *= .009) and CNV burden (*p *= .107) than lower sites (Figure S1D–F). The proportion of prevalent signatures was further analysed, and no significant difference was observed. However, signature 4, associated with smoking and likely due to tobacco mutagens, was more frequently found in tumours from upper sites compared to lower sites (8/14, 57.14% vs. 1/13, 7.69%; *p *= .013). Additionally, patients with signature 4 exhibited a higher response rate to the combination therapy than those without (7/9, 77.78% vs. 8/18, 44.44%; *p *= .217). And patients with the defective DNA mismatch repair‐related signature 15 was less likely to response to atezolizumab and bevacizumab combination therapy than those without signature 15 (2/8, 25.0% vs. 13/19, 68.4%; *p *= .087).

*KIT* and *NRAS* were the most prevalent driver genes in the ITT population, each occurring at a rate of 14.0% (6/43). Among patients with a *KIT* mutation, the ORR was 50% (3/6), while those with an *NRAS* mutation achieved an ORR of 83.3% (5/6). For genes associated with antigen presentation, the mutations enriched in non‐responders and mainly belong to major histocompatibility complex (MHC) class II antigen (Figure 2A). The other frequently mutated genes with mutation frequency above 10% were listed in Figure S2, and *PKHD1L1*, which expression was determined to be positively associated with OS and immune cell infiltration in cutaneous melanoma,^28^ was found to be more frequently mutated in responders than in non‐responders (33.3%, 5/15 vs. 8.3%, 1/12, *p *= .182). In the CNV profile, amplifications of *IL‐7*, *STK3* and *TP53INP1* were more frequent in responders (Figure 2A). Interestingly, these genetic aberrations were also more prevalent in upper sites compared to lower sites. At the arm‐level analysis, differential responses to therapy were observed: amplification of the 12q14.1 locus, which harbours *CDK4* were more frequent in non‐responders, and deletion of the 9q21.3 locus, which includes *CDKN2A*, were more frequent in responders (Figure 2B). Notably, the CNV profiles differed between upper and lower sites (Figure 2C).

The whole exome variant type and classification were similar between responders and non‐responders (Figure 2D). We used PyClone to compare the differences in intratumour heterogeneity between the responder group and the non‐responder group. Responders and non‐responders had different sets of clones and subclones, with few shared gene clones between them (Figure 2E,F). Responders had less intratumour heterogeneity, as their main clone frequency was significantly higher than that observed in non‐responders. Enrichment analysis showed that in responders, genes from the main clones were significantly involved in the PI3K‐Akt pathway, while genes from subclones were involved in the MAPK pathway (Figure 2G). In non‐responders, this pattern was reversed: the PI3K‐Akt pathway was more active in subclones, and the MAPK pathway in main clones. Additionally, sensory perception‐related genes were more prominent in the main clones of non‐responders. These differences between the two groups suggest the clonal evolution patterns and intratumour heterogeneity may influence the varying response to atezolizumab and bevacizumab combination therapy.

### 2.3 *NRAS* mutant mucosal melanoma exhibits enhanced angiogenesis

**FIGURE 3:**
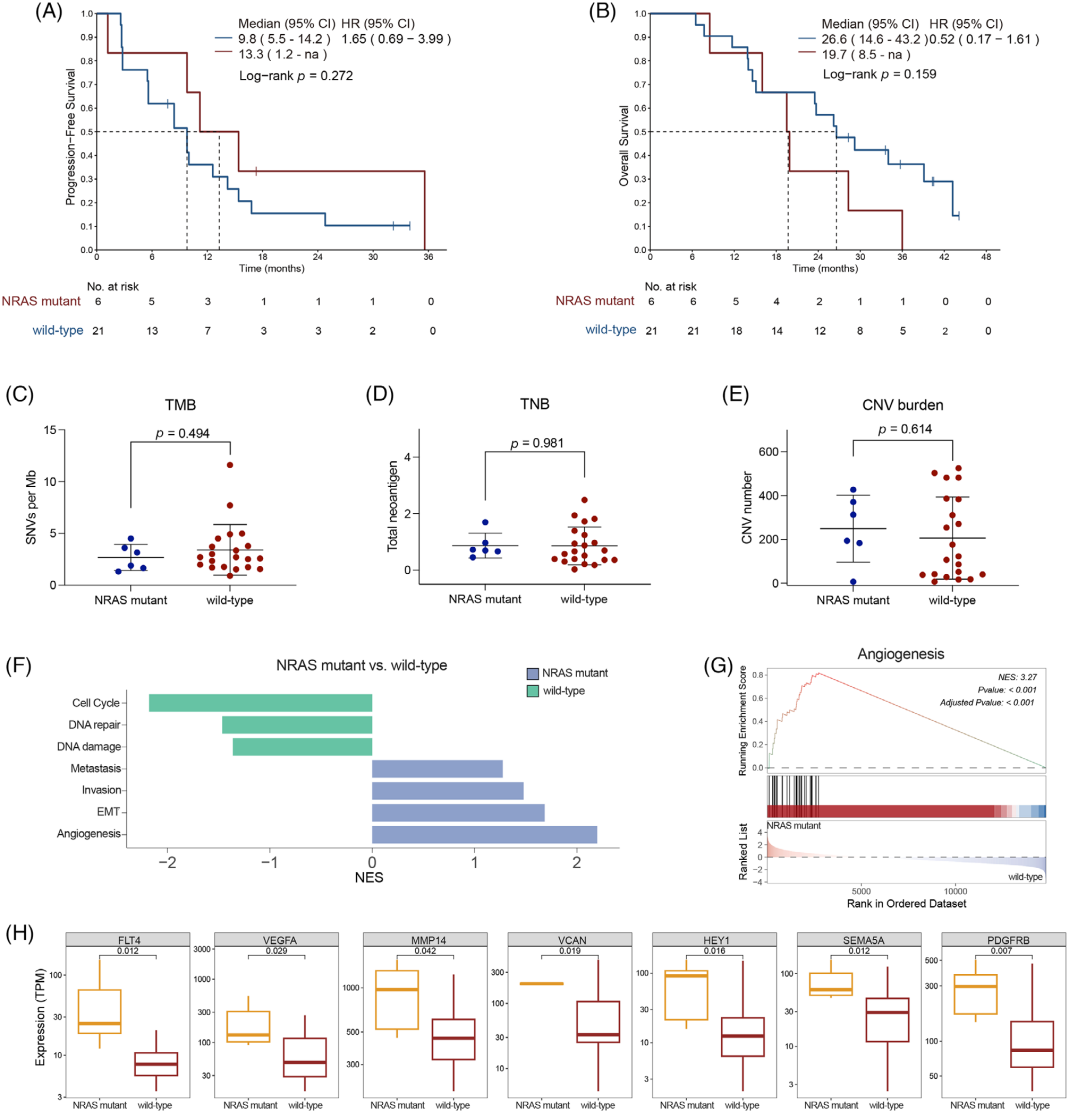
Mucosal melanoma with *NRAS* mutation exhibits elevated angiogenic characteristics. (A, B) Progression‐free survival (PFS) (A) and overall survival (OS) (B) of 43 intention‐to‐treat (ITT) patients in relation to *NRAS* mutations. (C–E) Genomic variances between groups stratified by *NRAS* mutation status. (C) Tumour mutation burden (TMB), (D) tumour neoantigen burden (TNB) and (E) copy number variation (CNV) burden are shown. (F) Bar plot showing enriched cancer single‐cell state atlas (cancerSEA) signatures in *NRAS* mutant and wild‐type groups. Green represents enrichment in the wild‐type group, and blue represents enrichment in the *NRAS* mutant group. (G) Gene set enrichment analysis (GSEA) plot of the angiogenesis gene set. (H) Differential expression of indicated genes in the angiogenesis pathway between *NRAS* mutant and wild‐type groups. CI, confidence interval; HR, hazard ratio.

Mucosal melanoma patients carrying *NRAS* mutations demonstrated a remarkable response rate to the combination therapy of atezolizumab and bevacizumab. Unlike the previous reports indicating shorter survival for *NRAS* mutant non‐cutaneous melanoma patients receiving ICIs compared to *NRAS* wild‐type patients,^29^ our analysis showed similar PFS between *NRAS* mutant and *NRAS* wild‐type patients (median: 13.3 months, 95% CI, 1.2–not reached vs. 9.8 months, 95% CI, 5.5–14.2; HR = 1.65, 95% CI, .69–3.99; *p *= .272; Figure 3A). OS was also similar between the two groups (median: 19.7 months, 95% CI, 8.5–not reached vs. 26.6 months, 95% CI, 14.6–43.2 months; HR = .52, 95% CI, .17–1.61; *p *= .159; Figure 3B). This suggests that incorporating anti‐angiogenic drug could extend the survival of mucosal melanoma patients with *NRAS* mutations to levels comparable to those without *NRAS* mutations.

To investigate the molecular factors influencing this response, we analysed the genomic and transcriptomic characteristics of *NRAS* mutant mucosal melanoma. The most frequently observed mutation site in responders was G12 (60%, 3/5), followed by G13 and Q61 (20%, 1/5 each). No notable differences were observed in TMB, TNB or CNV burden between *NRAS* mutant and *NRAS* wild‐type patients, indicating that the superior response to the combination therapy was not associated with the overall genomic aberration burden (Figure 3C–E).

Gene set enrichment analysis (GSEA) using the cancer single‐cell state atlas (cancerSEA) database revealed that angiogenesis, epithelial–mesenchymal transition (EMT), invasion and metastasis signatures were significantly enriched in *NRAS* mutant mucosal melanoma. In contrast, DNA damage, DNA repair and cell cycle signatures showed significant enrichment in *NRAS* wild‐type melanoma (Figure 3F). Among these pathways, the angiogenesis pathway was the most significantly enriched in the *NRAS* mutant group (*p* < .001, Figure 3G). Representative genes within the angiogenesis pathway, such as *FLT4* and *VEGFA*, exhibited higher expression in *NRAS* mutant patients than *NRAS* wild‐type patients (Figure 3H). These findings suggest that this enhanced therapeutic efficacy of combining ICIs with angiogenesis inhibitors may correlate with the more active angiogenesis pathway in mucosal melanoma patients with *NRAS* mutations.

### 2.4 Inflammatory cell infiltration, angiogenic status and p38 MAPK pathway activation associates with therapeutic response

**FIGURE 4:**
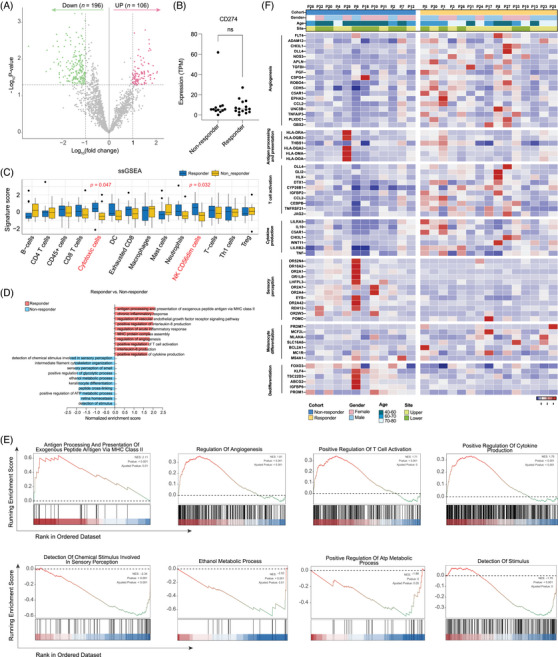
Transcriptomic signatures associated with the efficacy of atezolizumab plus bevacizumab therapy. (A) Volcano plot illustrating the number of differentially expressed genes between responders and non‐responders. (B) Expression of *CD274* in the two groups. ns, no significance. (C) Signature score of immune gene sets by single‐sample gene set enrichment analysis (ssGSEA). (D, E) GSEA was performed to identify different functional enrichment features between the two groups. (D) Bar plot depicting GSEA results. Red represents enrichment in responders, and blue represents enrichment in non‐responders. (E) GSEA plots of representative gene sets. NES, normalised enrichment score. (F) Heatmap representing the expression of genes in the indicated pathways.

Transcriptome sequencing was performed on 12 non‐responders and 15 responders, identifying a total of 302 differentially expressed genes (DEGs; Limma, |log2fold‐change|>1.0, *p* < .05; Figure 4A). The level of *CD274* (which encodes PD‐L1) was similar between two groups (Figure 4B). Immune cell infiltration assessed through single‐sample GSEA (ssGSEA) analysis revealed that the cytotoxic cells and CD56^dim^ NK cells were less infiltrated in non‐responders than in responders (Figure 4C). Unbiased GSEA indicated that angiogenesis and most inflammation‐related gene sets were significantly more enriched in responders. In contrast, gene sets related to sensory perception and metabolism were more enriched in non‐responders (Figure 4D,E). Heatmaps depict the expression of representative genes in each indicated pathway (Figure 4F). In addition to a more pronounced angiogenesis and pro‐inflammation profile, genes related to differentiation were more abundant in responders, while genes associated with de‐differentiation were more prevalent in non‐responders.

**FIGURE 5:**
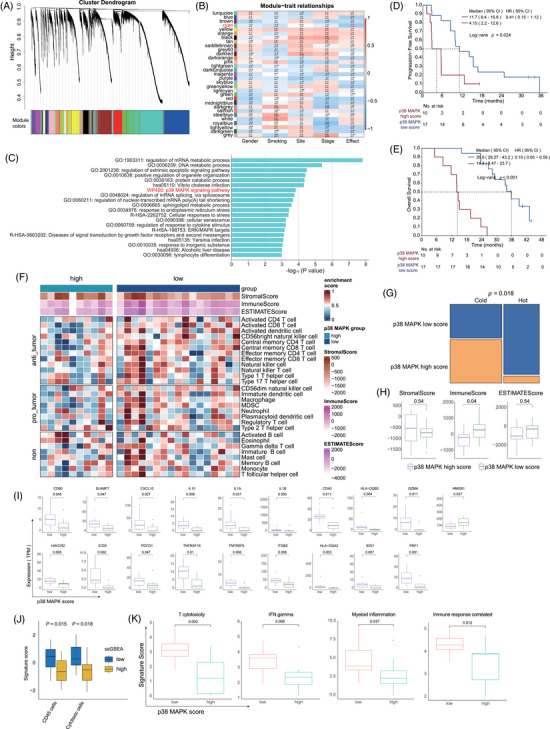
Identification of the p38 MAPK pathway related to the efficacy of atezolizumab plus bevacizumab therapy. (A) Hierarchical clustering dendrogram of modules delineated by weighted gene co‐expression network analysis (WGCNA), with each branch representing the colour categorisation of modules in the initial colour band below the dendrogram. (B) Heatmap of the module–trait correlations. Each cell indicates the Pearson correlation coefficient and corresponding *p* value. (C) Gene Ontology (GO) analysis representing the top‐ranked pathways based on the eigengenes. (D, E) Kaplan–Meier plots of progression‐free survival (PFS) (D) and overall survival (OS) (E) in patients stratified by p38 MAPK pathway expression score. (F) Heatmap of StromalScore, ImmuneScore, ESTIMATEScore and immune‐infiltrating cells in high and low p38 MAPK groups. (G) Bar chart representing the prevalence of cold/hot tumour in patients with high and low p38 MAPK score. (H) Box plot comparing StromalScore, ImmuneScore and ESTIMATEScore between groups categorised by p38 MAPK score. (I) Differential expression of co‐stimulation and antigen presentation‐relate genes between the two groups. (J) Single‐sample gene set enrichment analysis (ssGSEA) signature scores of CD45+ cells and cytotoxic cells. (K) T cytotoxicity, interferon (IFN)‐gamma, myeloid inflammation and immune response correlated signatures between high and low p38 MAPK group.

We investigated whether previously reported gene sets associated with immunotherapy response in cutaneous melanoma could distinguish between responders and non‐responders. However, none of these gene sets were effective in differentiating the two groups (Figure S3A). Additionally, there are no significant differences between non‐responders and responders in signature scores such as T cytotoxicity,^26^ interferon (IFN)‐gamma,^30^ myeloid inflammation^26^ and immune response correlated signature^31^ (Figure S3B). Therefore, weighted gene co‐expression network analysis (WGCNA) was carried out to identify clusters significantly correlated with prognosis. Significant spearman correlations of the eigengene values with clinical efficacy were identified and selected for further analysis (Figure 5A,B). These selected eigengenes underwent biological pathway enrichment analysis (PEA), revealing a focus on p38 MAPK pathway (Figure 5C). Patients with a low p38 MAPK score exhibited significantly longer mPFS (11.7 months, 95% CI, 8.4–16.8 vs. 4.15 months, 95% CI, 2.2–12.6; HR = .41, 95% CI, .15–1.12; *p *= .024; Figure 5D) and mOS (36.0 months, 95% CI, 28.27–43.2 vs. 14.3 months, 95% CI, 8.47–23.7; HR = .16, 95% CI, .05–.59; *p *< .001; Figure 5E) in comparison to those with a high p38 MAPK score. This OS benefit was consistent among both responders and non‐responders (Figure S4). Furthermore, lower p38 MAPK values were associated with a ‘hotter’ tumour immune microenvironment, characterised by higher infiltration of anti‐tumour immune cells and higher immune scores (Figure 5F–H). Patients with a low p38 MAPK score had notably higher levels of co‐stimulation and antigen presentation‐related genes than those with a high p38 MAPK score (Figure 5I). ssGSEA analysis confirmed that p38 MAPK low group was characterised by higher infiltration of CD45+ and cytotoxic cells (Figure 5J, *p* < .05). Interestingly, the aforementioned scores for T cytotoxicity, IFN‐gamma, myeloid inflammation and immune response correlated signatures were higher in the p38 MAPK low group compared to the p38 MAPK high group (Figure 5K).

### 2.5 Gene regulatory networks revealed by methylation and transcriptome profiles identify *SMAD2* association with therapeutic response

**FIGURE 6:**
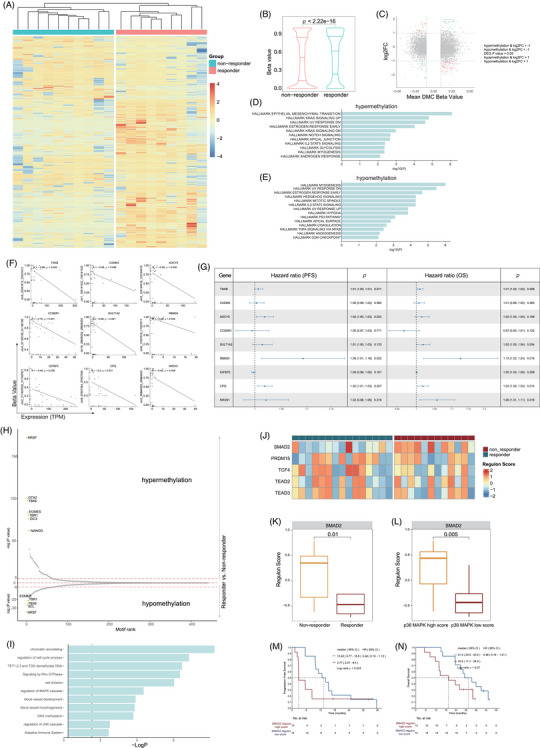
Methylation profile and gene regulatory networks analysis between responders and non‐responders. (A) Unsupervised hierarchical clustering of responders and non‐responders responders based on targeted bisulphite sequencing (TBS). (B) Violin plot showing the beta values in the two groups. (C) Integrating CpG methylation and mRNA expression in a scatter plot. The *y*‐axis displays gene expression differences (log2fold‐change), while the *x*‐axis shows the mean differentially methylated CpG (differential methylation cytosine [DMC]) beta values. Red dots represent hypermethylated and significantly down‐expressed genes in responders, orange dots represent hypomethylated and significantly up‐expressed genes in responders, blue dots represent hypermethylated but significantly up‐expressed genes in responders and green dots represent hypomethylated but significantly down‐expressed genes in responders. (D, E) Hallmark enrichment analysis representing the top‐ranked pathways based on hypermethylated genes (D) and hypomethylated genes (E) in responders compared to non‐responders. (F) Linear analysis of beta values and expression levels identifying genes those methylation levels are significantly negatively correlated with their expression level. (G) Forest plot of hazard ratio for progression‐free survival (PFS) and overall survival (OS) according to selected gene expression. (H) Differentially methylated transcription factor binding domains in responder versus non‐responder. (I) Bar plot showing pathway enrichment based on differentially methylated transcription factors. (J) Heatmap deciphering the regulon activity score of identified specific gene regulatory networks between responders and non‐responders. (K, L) Box plot showing *SMAD2* regulon scores between groups categorised by treatment response (K) and p38 MAPK score (L). (M, N) Kaplan–Meier plots of PFS (M) and OS (N) in patients stratified by *SMAD2* regulon score.

Methylation profiles were derived from targeted bisulphite sequencing (TBS) data obtained from nine responders and 10 non‐responders. Unsupervised hierarchical cluster analysis based on 7710 differential methylation cytosines (DMCs) effectively separated responders and non‐responders into two distinct clusters (Figure 6A), and the beta values of responders were significantly greater than non‐responders (Figure 6B, *p* < .001), indicating that the methylome of responders differs from non‐responders, with responders displaying a higher frequency of gene hypermethylation. To explore the significance of DNA methylation in gene expression regulation, integration of DNA methylation patterns with gene expression profiles from patient samples was performed. The integrated analysis revealed 43 DEGs corresponding to 103 DMCs that exhibited an inverse correlation (Figure 6C, |log2FC|>1, |DMC beta value|>.1 and *p* < .05). Hallmark enrichment analysis revealed that EMT‐associated genes were hypermethylated, while angiogenesis‐associated genes were hypomethylated in responders (Figure 6D,E). In the whole mucosal melanoma cohort including responders and non‐responders, the methylation levels of nine genes showed a statistically significant negative correlation with their expression level: extracellular matrix glycoprotein encoding gene *TNXB*, cell adhesion molecule *CADM3*, adenylyl cyclase enzymes encoding gene *ADCY5*, coiled‐coil serine rich protein *CCSER1*, sulfotransferase encoding gene *SULT1A2*, RNA‐binding protein *RBM24*, insulin‐like growth factor binding protein *IGFBP2*, carboxypeptidase *CPQ* and neurexin encoding gene *NRXN1* (Figure 6F, *p* < .05). Among these, the expression of *ADCY5*, *RBM24* and *CPQ* was negatively correlated with PFS, and the expression of *TNXB*, *SULT1A2*, *RBM24*, *CPQ* and *NRXN1* was negatively correlated with OS (Figure 6G).

Methylation at specific genomic loci can impede transcription factor (TF) binding and modulate the regulation of target gene expression,^32^ therefore, we examined the differential methylation of TF‐binding motifs (Figure 6H). PEA suggested that these TFs regulate genes associated with cell cycle, cell division, MAPK pathway, blood vessel development and morphogenesis, DNA methylation and adaptive immune system (Figure 6I). Next, we used regulon analysis to explore specific gene regulatory networks potentially impacting treatment response. By comparing the regulon scores, we examined the crucial regulon of each sample, we identified *SMAD2*, *PRDM15*, *TCF4*, *TEAD2* and *TEAD5* were the top regulons in the cohort (Figure 6J). The regulon score of *SMAD2* was notably lower in the responder group than the non‐responder group (*p *= .01, Figure 6K), and lower in P38 MAPK low group compared to the P38 MAPK high group (*p *= .005, Figure 6L). Furthermore, patients with lower *SMAD2* regulon scores had longer mPFS (13.4 months, 95% CI, 9.77–16.8 vs. 2.77 months, 95% CI, 2.57–8.4; HR = .46, 95% CI, .19–1.12; *p* = .043; Figure 6M) and mOS (31.3 months, 95% CI, 23.5–43.2 vs. 19.5 months, 11.7–34.0; HR = .48, 95% CI, .19–1.21; *p* = .07; Figure 6N) than those with higher *SMAD2* regulon scores.

## 3 DISCUSSION

While ICIs have revolutionised melanoma treatment, accumulating clinical studies consistently demonstrate that outcomes of mucosal melanoma are worse compared to those for cutaneous melanoma. In the KEYNOTE‐001, 002 and 006 trials, 84 advanced mucosal melanoma patients administrated with anti‐PD‐1 monotherapy exhibited an ORR of 19%, with mPFS and mOS of 2.8 months and 11.3 months, respectively.^13^ An international retrospective study of 548 advanced mucosal melanoma showed that those received anti‐PD‐1 monotherapy (*n* = 348) had an ORR of 29%, mPFS of 5 months and mOS of 19 months. In contrast, patients receiving dual ICIs (*n* = 197) had an ORR of 31%, mPFS of 4 months and mOS of 21 months. Except for nasal/oral primary sites, dual ICIs showed no advantage over monotherapy in other primary sites.^33^

Our clinical study showed that the combination of atezolizumab and bevacizumab exhibits therapeutic efficacy in advanced mucosal melanoma, with an ORR and median PFS surpassing those observed in monotherapy and dual ICIs treatments. After 40.3 additional months of follow‐up, the mPFS was 8.4 months, and the mOS was 23.7 months. These outcomes align with another study using toripalimab combined with axitinib, which reported an ORR of 48.3%, mPFS of 7.5 months and mOS of 20.7 months.^34^ These studies suggest that ICI and anti‐angiogenesis therapy combination is a promising option for mucosal melanoma, offering superior response rates and longer PFS than anti‐PD‐1 monotherapy and dual ICIs.

In this updated analysis, the survival advantage for patients with primary lesions from upper sites and the superior response rate of those with *NRAS* mutations was maintained. Through integrating genetic, transcriptomic and methylomic profiling of baseline tumours from patients enrolled in this trial, we identified molecular correlates of clinical response. Mucosal melanoma of upper sites exhibited a higher prevalence of smoking‐related signature 4, increased TNB and marginally higher CNV burden than those of lower sites. Newell et al. reported that ultra violet radiation (UVR)‐related signature was prone to be found in mucosal melanoma of upper sites, and age‐related signature was more present in those of lower sites.^10^ These observations suggest that distinct carcinogenic processes may drive mucosal melanoma at different anatomical sites, with melanomas at upper sites potentially subject to a higher mutagenic pressure. This could lead to the generation of more immunogenic neoantigens, rendering these tumours more susceptible to ICI‐based treatments. The differential prevalence of these signatures highlights the need for tailored therapeutic approaches that consider the specific mutational landscape and immune contexture of mucosal melanomas at different anatomical sites. Furthermore, the association of higher TNB and CNV burden with mucosal melanomas from upper sites provides additional biomarkers that could be used to stratify patients more accurately and predict their response to therapy.

Regarding *NRAS* mutation, their frequency did not vary significantly across melanoma subtypes, though mutation sites differed, with Q61 predominant in cutaneous and G12 in mucosal melanomas.^9^ The impact of *NRAS* mutation on ICI efficacy is controversial. Johnson et al. observed an ORR of 64% in *NRAS* mutant advanced cutaneous melanoma, attributed to higher mutational burdens and PD‐L1 positivity.^35^ In the retrospective study aimed to explore the effects of *BRAF* and *NRAS* mutations on the effectiveness of ICIs in 1764 patients with advanced cutaneous melanoma.^36^ They found no differences in response and survival between patients with *NRAS* mutations and those with both *BRAF* and *NRAS* wild‐type in settings of both anti‐PD‐1 monotherapy and dual ICIs. In contrast, Zhou et al. demonstrated that acral and mucosal melanoma patients with *NRAS* mutation treated anti‐PD‐1 monotherapy had shorter PFS and OS than patients without *NRAS* mutation.^29^

Our study observed an 83.3% response rate in *NRAS* mutant patients, a rate significantly higher than that observed in the overall cohort. However, no correlations were found between *NRAS* status and either TMB or *CD274* level in our study. A real‐world study by Tang et al. on anti‐PD‐1 antibody plus axitinib as first‐line treatment in advanced mucosal melanoma reported an ORR of 62.5% (5/8) among those with *NRAS* mutations, which is twice as high as that of the entire cohort (30.0%, 24/80).^37^ Both our study and that of Tang et al.’s study suggests that, unlike treatments with ICIs alone, the combination of anti‐angiogenic treatments exhibits a high response rate in *NRAS* mutant mucosal melanoma. Additionally, we found that atezolizumab and bevacizumab extended the PFS and OS of *NRAS* mutant patients to levels similar to those of wild‐type patients, and *NRAS* mutant mucosal melanoma exhibiting enhanced angiogenesis feature with elevated *VEGFA* expression. This hints at a link between *RAS* mutations and angiogenesis. Previous studies have shown that *HRAS* mutations can activate the PI3K/AKT/mTOR pathway, thereby promoting angiogenesis through the upregulation of VEGF and other pro‐angiogenic factors.^38^ Additionally, *NRAS* mutations have been demonstrated to drive abnormal angiogenesis in human endothelial cells.^39^ *GAB2*, found to be amplified in 30.6% of acral melanomas and 17.1% of mucosal melanomas,^40^ has been shown to induce tumour angiogenesis by regulating hypoxia inducible factor 1 subunit alpha (HIF‐1α) and VEGF expressions in *NRAS*‐driven melanoma.^41^ Consequently, we hypothesise that the superior response of *NRAS* mutant melanoma to ICI and anti‐angiogenic combination treatment may be due to its more pronounced abnormal angiogenesis, making it more susceptible to anti‐angiogenic treatment. Nevertheless, the influence of *NRAS* mutations on the effectiveness of anti‐angiogenic treatment and immunotherapy requires further validation in large‐scale prospective studies.

Our transcriptomic and methylation analyses identified of 302 DEGs and 7710 DMCs between the responder group and the non‐responder group. These notable differences in gene expression and methylation profiles highlight the complex molecular mechanisms underlying response to therapy. Notably, the absence of significant differences in *CD274* expression between responders and non‐responders suggests that PD‐L1 may not be a reliable sole predictor of response in this context, and underscores the necessity of exploring additional biomarkers and mechanisms that could more accurately predict combination therapeutic outcomes. The increased prevalence of cytotoxic cells and CD56dim NK cells in responders indicates a potent anti‐tumour immune environment in responders. GSEA analysis further revealed that responders exhibited enrichment in gene sets related to angiogenesis, IFN‐gamma response, antigen processing and presentation and T‐cell activation. The methylation analysis also found that methylation patterns related to angiogenesis and adaptive immune system was enriched in responders. This suggests that combination of anti‐angiogenic and anti‐PD‐1 treatment synergistically enhances the immune microenvironment's capacity to generate a potent anti‐tumour response by facilitating both T‐cell activation and modulating the tumour vasculature. The angiogenesis signature's prominence in responders underscores the critical role of vascular modulation in enhancing immunotherapy outcomes. Angiogenesis not only supports tumour growth by supplying nutrients and oxygen but also modulates the tumour microenvironment to evade immune surveillance. The anti‐angiogenic component of the treatment can disrupt tumour vasculature, improving immune cell infiltration and function.^18^

Our study utilised WGCNA to discover significant correlations between eigengene values and clinical efficacy. Through detailed biological PEA, we observed that patients with high p38 MAPK activity had notably shorter PFS and OS, and the OS benefit was consistent in both responders and non‐responders. Notably, elevated p38 MAPK levels were associated with a ‘colder’ tumour immune microenvironment, marked by reduced infiltration of key anti‐tumour immune cells. GSEA further indicated that pathways critical for an effective immune response were less active in the high p38 MAPK group. The blockade of p38 MAPK signalling was observed to decrease M‐MDSC infiltration in the liver, which showed a positive correlation with the proportion of CD8+ T cells and a negative association with tumour mass, thus inhibiting the progression of hepatocellular carcinoma.^42^ Additionally, inhibiting p38 could enhance the anti‐tumour effect of T cells in melanoma and improve the functionality of human tumour‐reactive and gene‐engineered T cells.^43^ Based on these findings, we suggest that targeting the p38 MAPK pathway may improve therapeutic outcomes by transforming ‘cold’ mucosal melanomas into ‘hot’ ones, thereby enhancing the effectiveness of ICIs and anti‐angiogenic combination treatments.

SMAD2, a key member of the SMAD family, acts as a critical mediator in the TGF‐β signalling pathway, influencing transcription downstream.^44^ It has been reported that SMAD2 is expressed at high level in most cancers, including melanoma, and is associated with the development and progression of tumours.^45^ Upon entering the nucleus, SMAD2 interacts with multiple TFs, affecting the expression of immune‐associated molecules such as IL‐17,^46^ BCL6.^47^ In our study, the higher SMAD2 regulon score in non‐responders compared to responders suggests a potential link between elevated SMAD2 activity and reduced treatment efficacy in mucosal melanoma. Furthermore, there is interplay between p38 MAPK pathway and the SMAD2 pathway, contributing to the fibrosis of blood vessels^48^, ^49^ and skin inflammation.^50^ These insights support our findings of positive association between the SMAD2 regulon score and the p38 MAPK score, warranting deeper exploration into the underlying mechanisms.

The application of multi‐omics technologies in this study has yielded important new insights into the treatment of mucosal melanoma. However, several challenges and limitations still impede the full realisation of its potential. In multi‐omics analyses, multiple comparisons can lead to a series of statistical and biological issues. The dependencies and correlations among different data layers (genomics, transcriptomics and proteomics) make multiple comparisons more complex, increasing the false positive rates due to many hypotheses testing across multiple data.^51^ While the sample size in this study was limited, we implemented rigorous evaluation methods to minimise the risk of false positives, ensuring precise matching of key parameters, such as *NRAS* mutation status and MAPK scores. Furthermore, we need to expand the sample cohort and conduct experimental validations, thus enhancing the understanding of tumourigenesis and improve the reliability of our findings.

In summary, our study demonstrates that combining ICIs with anti‐angiogenic therapy, specifically atezolizumab plus bevacizumab, offers promising improvements in response and survival in mucosal melanoma. This suggests a potential strategic advantage in the treatment of mucosal melanoma. Our findings also highlight the impact of anatomical site, *NRAS* mutation status, activity of SMAD2 and p38 MAPK pathway on treatment responses. Specifically, mucosal melanomas originating from upper sites and those harbouring *NRAS* mutations may more benefit from the combination therapy. Additionally, high activity of the p38 MAPK pathway was associated with a diminished immune response, indicating that targeting this pathway could potentially improve the efficacy of ICIs and anti‐angiogenic treatments. Our research underscores the multifactorial characteristics of response to cancer immune therapy, emphasising the necessity of integrating genomic, transcriptomic and epigenomic data to identify the determinants of treatment response. This comprehensive approach enhances our understanding of the mechanisms behind immune evasion and therapy resistance but also lays the groundwork for the need for more refined and efficient treatment approaches for mucosal melanoma. Additional studies are required to investigate the functional implications of these findings in larger and more diverse cohorts. Moreover, investigating the potential of incorporating these molecular signatures into predictive models for immunotherapy response holds promise for guiding treatment decisions and enhancing patient outcomes.

## 4 MATERIALS AND METHODS

### 4.1 Patients

Data from patients with unresectable or metastatic mucosal melanoma who received treatment with atezolizumab and bevacizumab in our previous clinical trial (NCT04091217) conducted between 2019 and 2020 were extracted and analysed (last follow‐up in August 2023).^35^ Atezolizumab was given intravenously at a dose of 1200 mg every 3 weeks, while bevacizumab was given intravenously at a dose of 7.5 mg/kg every 3 weeks. Radiological assessments were conducted by investigators following the RECIST 1.1. The ORR was determined as the percentage of patients who achieved complete response (CR) or partial response (PR) to the treatment. Patients were classified into two groups: responders (CR or PR) and non‐responders (stable disease, SD; or progressive disease, PD). PFS was measured as the time from the initiation of treatment to disease progression or the last follow‐up, while OS was calculated as the time from the initiation of treatment to the last follow‐up or death.

### 4.2 Tumour specimens and profiling

Formalin fixed paraffin‐embedded (FFPE) tumour samples and corresponding peripheral blood mononuclear cells (PBMCs) were collected. FFPE samples underwent DNA and RNA co‐extraction, and paired peripheral white blood cells (WBCs) genomic DNA was used as a control for WES. All participants given written informed consent before any study‐related procedures were conducted. The study adhered to the principles set forth in the Declaration of Helsinki.

### 4.3 WES data processing

#### 4.3.1 DNA extraction and sequencing

DNA was extracted from FFPE and PBMCs, then fragmented into 200–300 bp using the Covaris S220 ultrasonic system. The library was constructed with a custom 53 M‐length capture probe provided by Integrated DNA Technologies (IDT). Captured libraries were subsequently sequenced in pair‐end reads of 100 bp in length by the DNBSEQ‐T7RS platform (BGI).

#### 4.3.2 Data pre‐processing and quality control

The raw data from DNBSEQ‐T7RS sequencing underwent initial filtering to eliminate low‐quality reads and those adaptor containing sequences. Subsequently, the reads were aligned to the reference human genome (hg19), with the Burrows‐Wheeler Aligner (BWA) aligner employed for mutation calling, followed by the application of several filtering procedures on the identified mutations. (1) Synonymous variants were excluded. (2) Variants with fewer than five high‐quality sequencing reads (mapqthres > 30, baseqthres > 30) for FFPE DNA, fewer than two high‐quality sequencing reads for PBMCs DNA were filtered out. (3) Mutations with a variant allele fraction (VAF) of less than 5% were filtered out.

#### 4.3.3 Mutational signature analysis

The mutation signatures of somatic single base substitutions across all samples were analysed using deconstructSigs, which utilised 30 COSMIC signatures included in the package.

#### 4.3.4 Copy number variations analysis

The integer copy numbers were estimated with CNVkit. The ratio of normalised coverage depth between tumour and normal WES samples was computed for each exome capture probe (log2 copy ratio of ±.1). Amplifications and deletions were defined using threshold of .848 and −.737, respectively. The distribution of coverage depth ratios was plotted for each tumour/normal pair. Samples or chromosomes exhibiting high variation in coverage ratio across the chromosomes were manually excluded from further analysis based on the coverage depth ratio plots. Regions of significant amplification and deletion were identified using GISTIC 2.0 with default parameters. Events with a *q*‐value less than .1 were considered statistically significant.

#### 4.3.5 Clonality evaluation

Tumour purity and genomic ploidy were evaluated with ABSOLUTE (version 1.2), incorporating analyses of cancer cell fractions and allele‐specific CNVs in each sample. PyClone (v0.13.1) was used to analyse the clonal population structure of tumour samples from each patient.

### 4.4 RNA‐seq data processing

#### 4.4.1 RNA extraction and sequencing

Tumour RNA was reverse‐transcribed into cDNA using Ribo‐SPIA Technology (NuGEN). The construction of the mRNA library, sequencing and QC of the FASTQ data were carried out as described in previous reports. Reads of low‐quality reads and containing adaptor containing sequences were filtered out. These reads were then aligned to the hg19 with HISAT. Following this, transcript assembly was conducted with StringTie (v1.2.3) to create comprehensive gene expression profiles from the RNA‐seq data.

#### 4.4.2 Differential expression gene analysis

Gene differential expression analysis between different groups was conducted using Limma software. Significantly DEGs were identified by applying thresholds of |log2fold‐change|>1 and a false discovery rate (FDR) < .05.

#### 4.4.3 Gene set enrichment analysis

GSEA was conducted using the R package clusterProfiler. The analysis utilised gene sets from the KEGG, Hallmark, Gene Ontology (GO), Reactome collections from the Molecular Signature Database to identify enriched pathways. The cancerSEA was employed to identify the enriched pathways related to cancer states. The ssGSEA applied a deconvolution method to analyse 24 immune cell types involved in immune responses. The immune cell infiltration levels were assessed using ssGSEA method with GSVA package.

#### 4.4.4 Weighted gene co‐expression networks analysis

To explore genes and pathways correlated with clinical response and non‐response, WGCNA was performed on the R package WGCNA with default parameter. The significant spearman correlation of the eigengenes values with clinical efficacy was selected, and the selected eigengenes were subjected to biological pathway enrichment. We selected 17 genes (MAPK14, MAX, MAP3K5, TGFBR1, ACTG1, ELK3, GIPR, P2RX1, PPARA, ADAM17, SARM1, APAF1, BCKDHB, VPS41, LRP11, TTC5, IL7) from the P38 MAPK pathway and applied the GSVA algorithm for quantitative scoring, and an optimal cut‐off score was determined by maximising the Youden index. Values exceeding the cut‐off are defined as P38 MAPK high score, while values below the cut‐off are defined as P38 MAPK low score.

### 4.5 Methylome analysis

#### 4.5.1 Targeted bisulphite sequencing

DNA of tumour tissues was subjected to bisulphite treatment to facilitate the construction of a sequencing library. TBS was conducted with SeqCap Epi 4M CpGiant probes from Roche using Illumina HiSeq platform (Illumina). The raw data were demultiplexed using bcl2fastq and subjected to quality trimming using Trimmomatic. The high‐quality reads obtained were aligned to the hg19 using the Bismark bisulphite aligner.

#### 4.5.2 Differential methylation cytosine and pathway enrichment analysis

The AsTair software (https://bitbucket.org/bsblabludwig/astair/src/master/) was used to detect methylation sites. The methylation levels at individual cytosine sites were quantified using beta values, defined as the ratio of methylated cytosine counts to the total number of cytosine counts at that site. The DMCs were analysed using the methylKit R package with default parameters set as methylation levels greater than .1 and CpG coverage of at least 10. Additional data filtering involved the use of the 2D‐KS and Mann–Whitney *U*‐tests, with significance defined as a threshold of *p* < .05. To manage the FDR, the Benjamini–Hochberg method was applied to adjust for multiple testing. ChIPseeker R package was utilised to annotate and visualise DMC gene coverage over chromosomes. The GO enrichment analysis was conducted using metascape with a *p*‐value of .01 for both the hyper‐ and hypomethylated DMC genes.

#### 4.5.3 Regulon analysis

We used the HOMER (http://homer.ucsd.edu/homer/) software to find motifs between 50 bp upstream and downstream of the methylation site with the findMotifsGenome.pl command. Subsequently, the R package RTN was employed to reconstruct transcriptional regulatory networks (regulons) related to the response.

### 4.6 Statistics

Survival differences between groups were assessed using the Kaplan–Meier method with the log‐rank test. Categorical variables were compared using either the Fisher's exact test or chi‐square test, while continuous variables were assessed with the Wilcoxon Mann–Whitney *U*‐test. Statistical significance for the unpaired two‐tailed Student's *t*‐test was defined as a *p*‐value <.05. All statistical analyses and visualisations were carried out using R software (version 4.1.2).

## AUTHOR CONTRIBUTIONS

Lu Si, Lili Mao and Yu Chen made substantial contributions to the study conception and design. Jie Dai, Tianxiao Xu, Meiyu Fang, Jing Lin, Jun Cao, Xue Bai, Caili Li, Xiaoting Wei, Junjie Gu, Jun Guo, Yu Chen, Lili Mao and Lu Si were involved in patient recruitment and data acquisition. Jie Dai, Tianxiao Xu, Lifeng Li, Yaoyao Liu, Xuan Gao and Xuefeng Xia did the analysis and interpretation of data, Jie Dai, Tianxiao Xu and Lifeng Li also contributed to manuscript preparing, review and editing. Lu Si, Lili Mao and Yu Chen were responsible for study supervision.

## CONFLICT OF INTEREST STATEMENT

The authors declare no conflicts of interest.

## ETHICS STATEMENT

This study was approved by Peking University Cancer Hospital institutional review board (2019YW90). Prior to any study procedures, patients provided consent in accordance with an IRB‐approved protocol.

## Supporting information



Supporting Information

Supporting Information

Supporting Information

Supporting Information

Supporting Information

## Data Availability

The human sequence data generated in this study are not publicly available due to patient privacy requirements but are available upon reasonable request from the corresponding author. Other data generated in this study are available within the article and its Supporting Information data files.
