# Supplementary figures and images for: Atezolizumab plus bevacizumab in patients with unresectable or metastatic mucosal melanoma: 3‐year survival update and multi‐omics analysis
Source: Clin Transl Med. 2025 Jan 5;15(1):e70169. doi: 10.1002/ctm2.70169 (PMC11702371; doi:10.1002/ctm2.70169)

# Supplementary Fig. S1

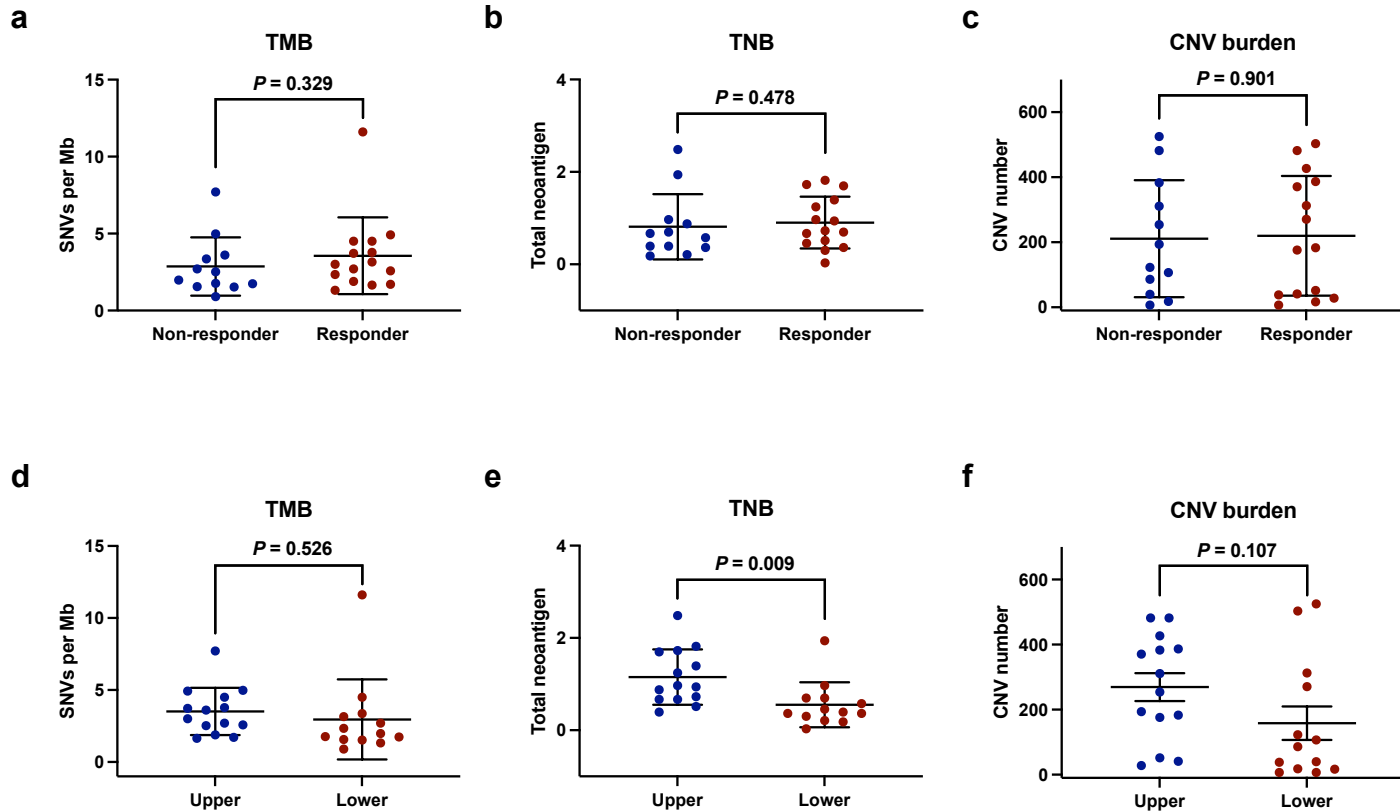

Supplement: Supplementary file 1 — Supporting Information [file CTM2-15-e70169-s005.pdf]

Supplementary Fig. S3

a

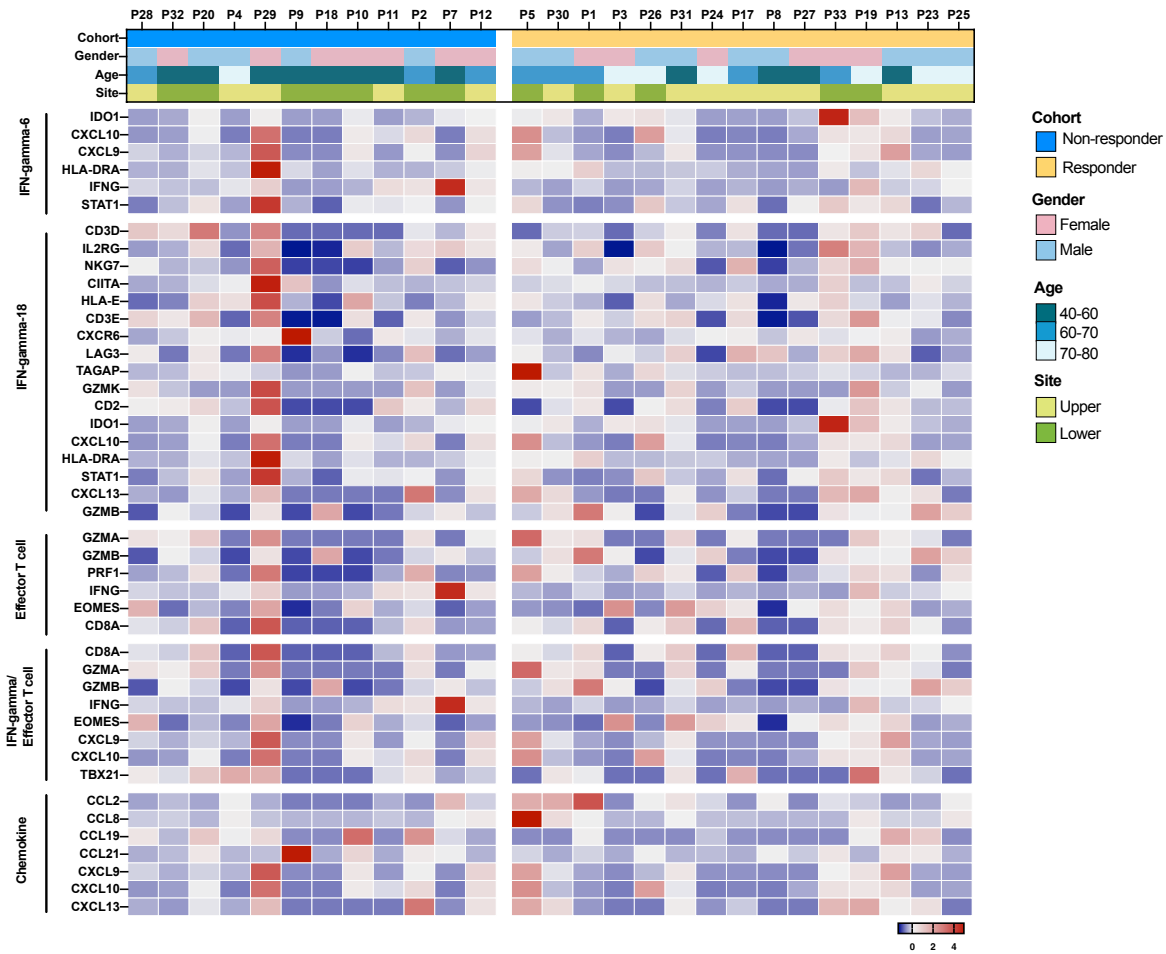

b

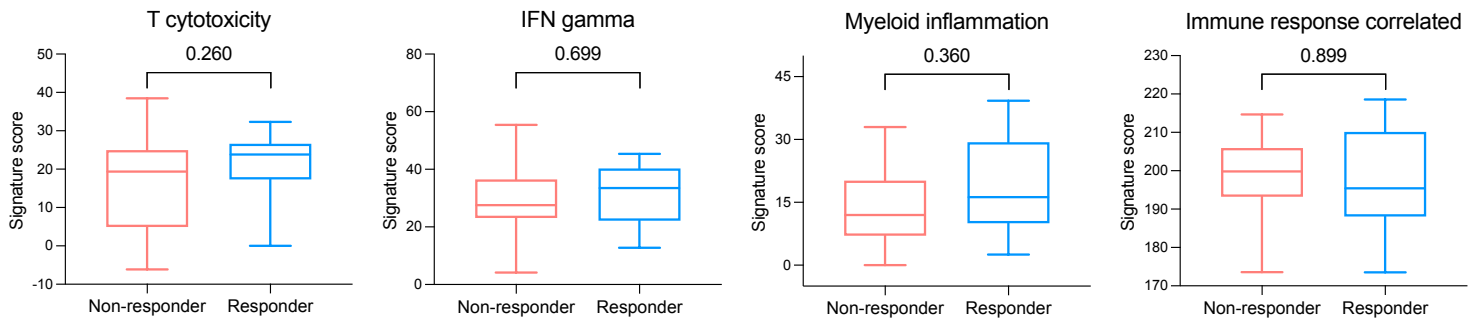

Supplement: Supplementary file 3 — Supporting Information [file CTM2-15-e70169-s001.pdf]

Supplementary Fig. S4

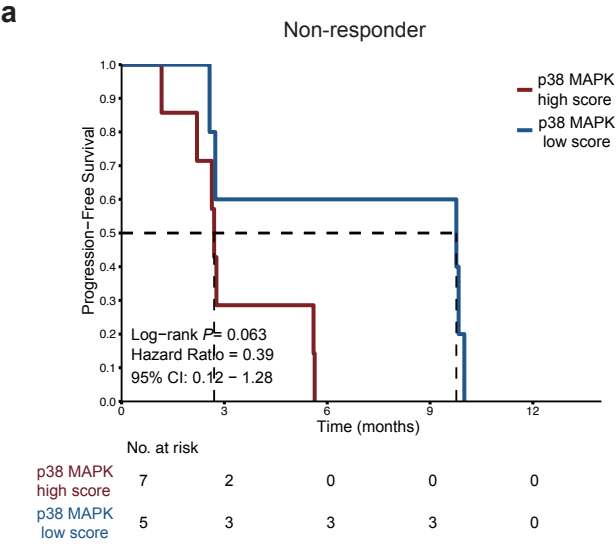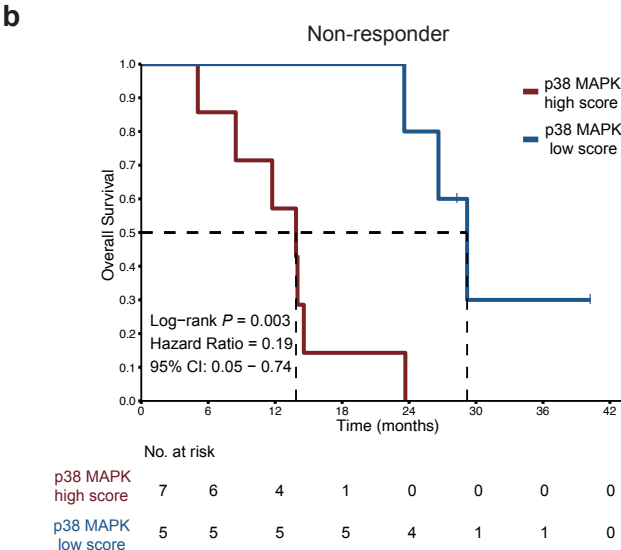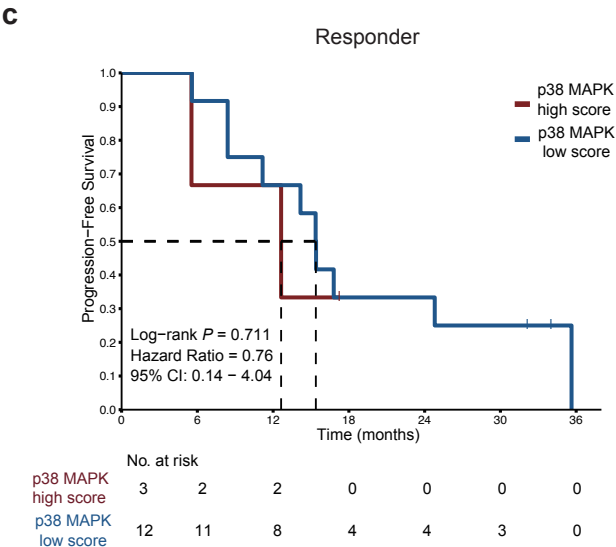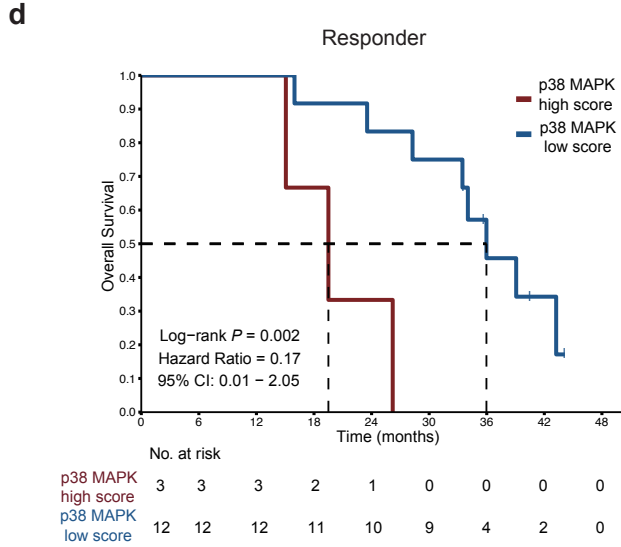

Supplement: Supplementary file 4 — Supporting Information [file CTM2-15-e70169-s002.pdf]
