# Supplementary material for: Atezolizumab plus bevacizumab in patients with unresectable or metastatic mucosal melanoma: 3‐year survival update and multi‐omics analysis
Source: Clin Transl Med. 2025 Jan 5;15(1):e70169. doi: 10.1002/ctm2.70169 (PMC11702371; doi:10.1002/ctm2.70169)

Supplementary Fig. S2

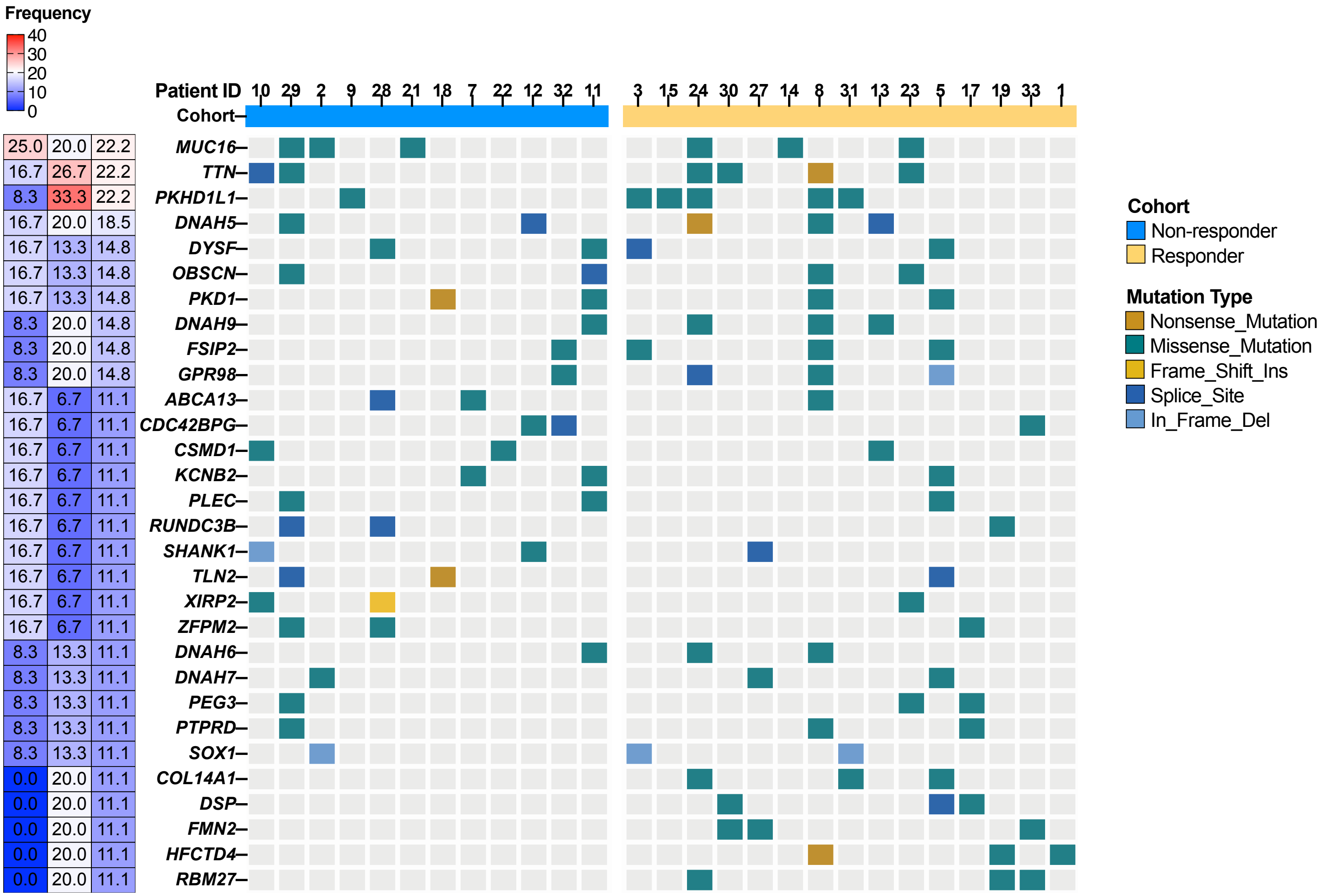

Supplement: Supplementary file 2 — Supporting Information [file CTM2-15-e70169-s004.pdf]
