# Supplementary material for: Atezolizumab plus bevacizumab in patients with unresectable or metastatic mucosal melanoma: 3‐year survival update and multi‐omics analysis
Source: Clin Transl Med. 2025 Jan 5;15(1):e70169. doi: 10.1002/ctm2.70169 (PMC11702371; doi:10.1002/ctm2.70169)
Supplement: Supplementary file 5 — Supporting Information [file CTM2-15-e70169-s003.docx]

| Supplementary Table S1. Patient demographics and baseline characteristics of the intention-to-treat population (N=43). | |
| --- | --- |
| Characteristics |  |
| Age, years |  |
| Median (range) | 61 (33–73) |
| Sex, n (%) |  |
| Male | 21 (48.8) |
| Female | 22 (51.2) |
| LDH, n (%) |  |
| Normal | 33 (76.6) |
| Elevated | 10 (23.3) |
| ECOG PS, n (%) |  |
| 0 | 32 (74.4) |
| 1 | 11 (25.6) |
| Primary site, n (%) |  |
| Head-neck | 15 (34.9) |
| Gastrointestinal | 17 (39.5) |
| Urogenital | 11 (25.6) |
| Baseline sum of longest diameter (mm) |  |
| Median (range) | 36.3 (10.0–217.0) |
| Disease stage, n (%) |  |
| III | 19 (44.2) |
| IV | 24 (55.8) |
| *BRAF*, n (%) |  |
| Wild-type | 42 (97.7) |
| Mutant | 1 (2.3) |
| *NRAS*, n (%) |  |
| Wild-type | 37 (86.0) |
| Mutant | 6 (14.0) |
| *KIT*, n (%) |  |
| Wild-type | 36 (83.7) |
| Mutant | 6 (14.0) |
| Unknown | 1 (2.3) |

ECOG PS, Eastern Cooperative Oncology Group performance status; LDH, lactate

526 dehydrogenase.
